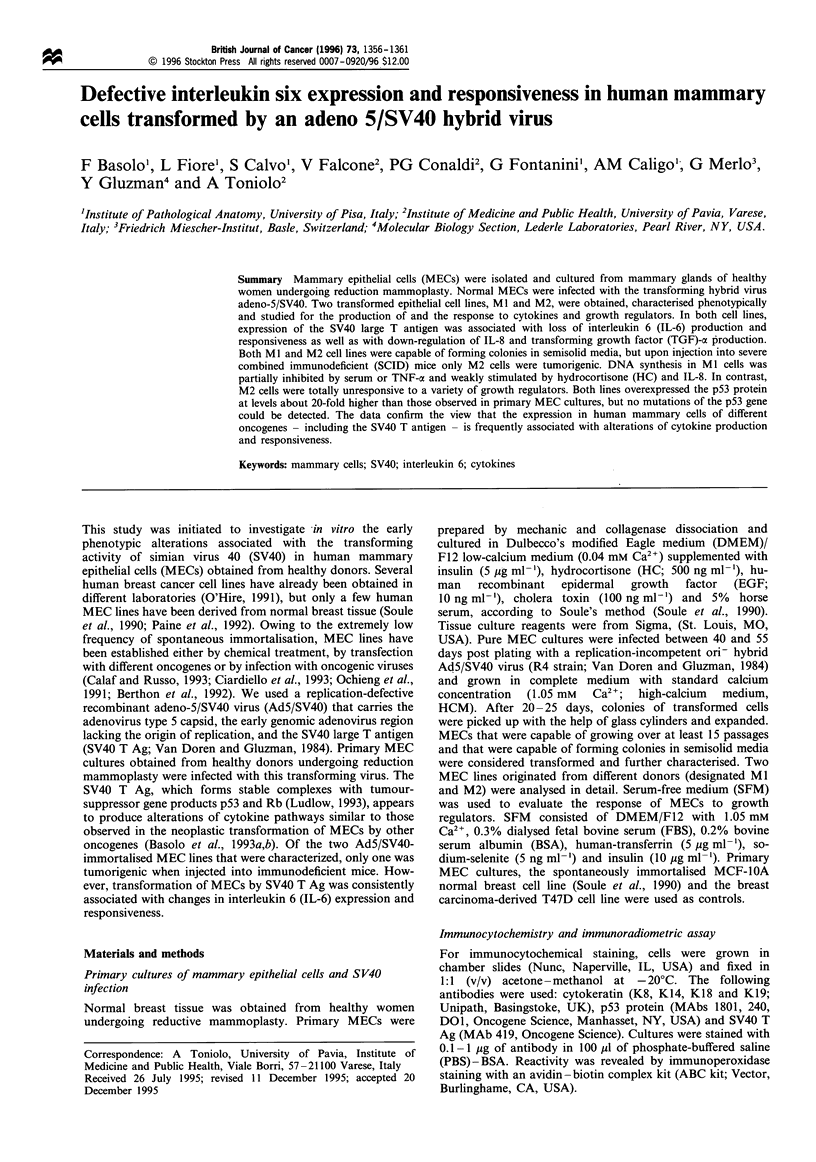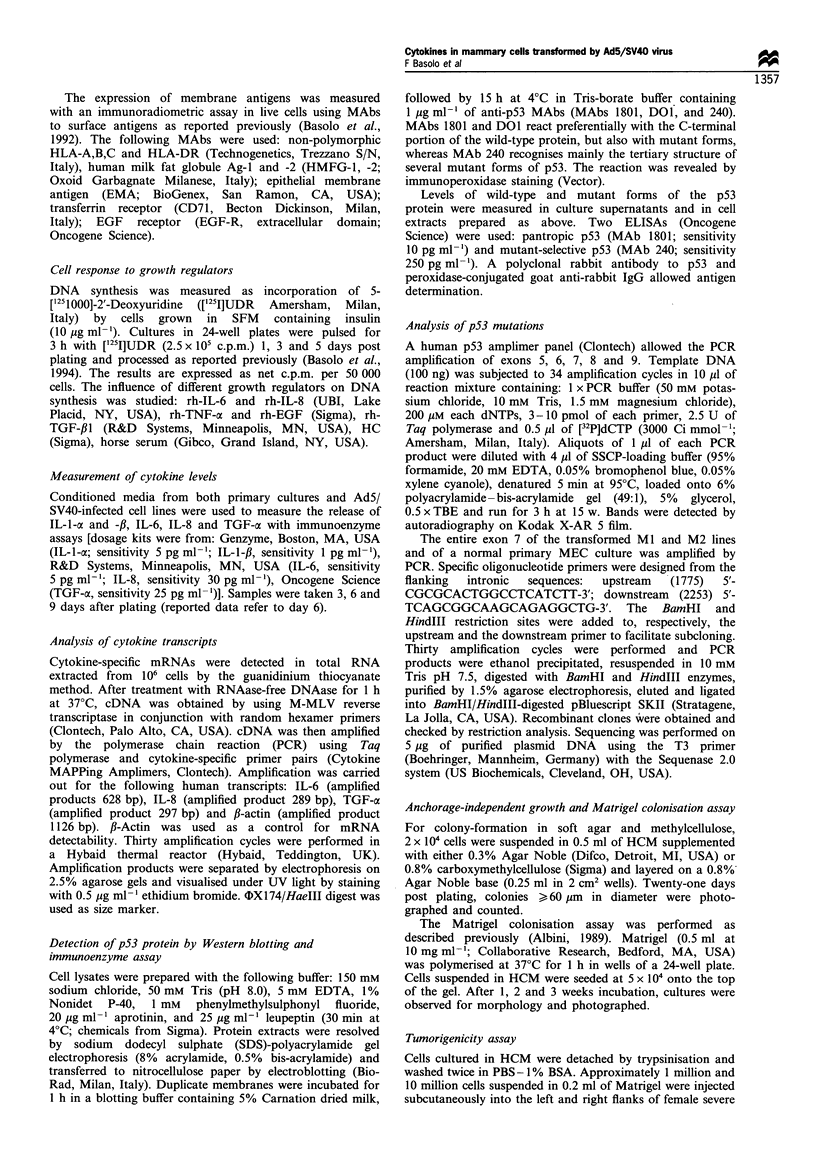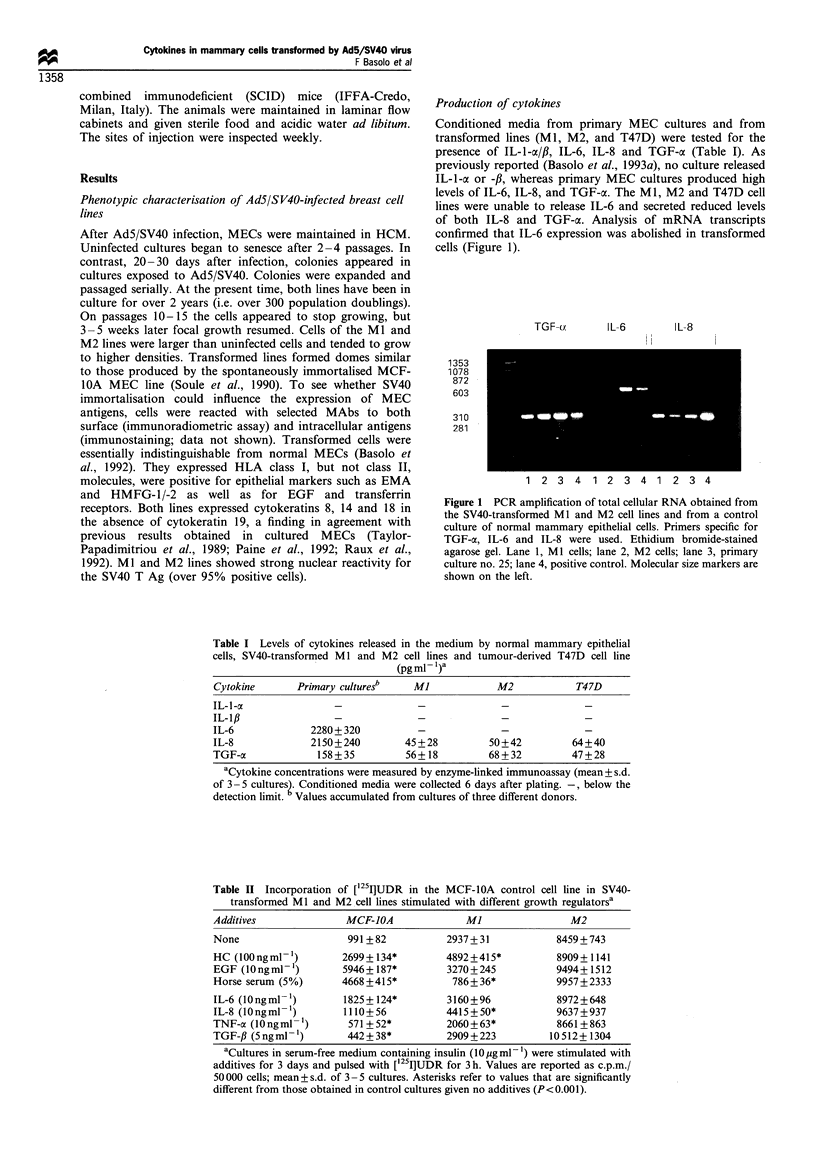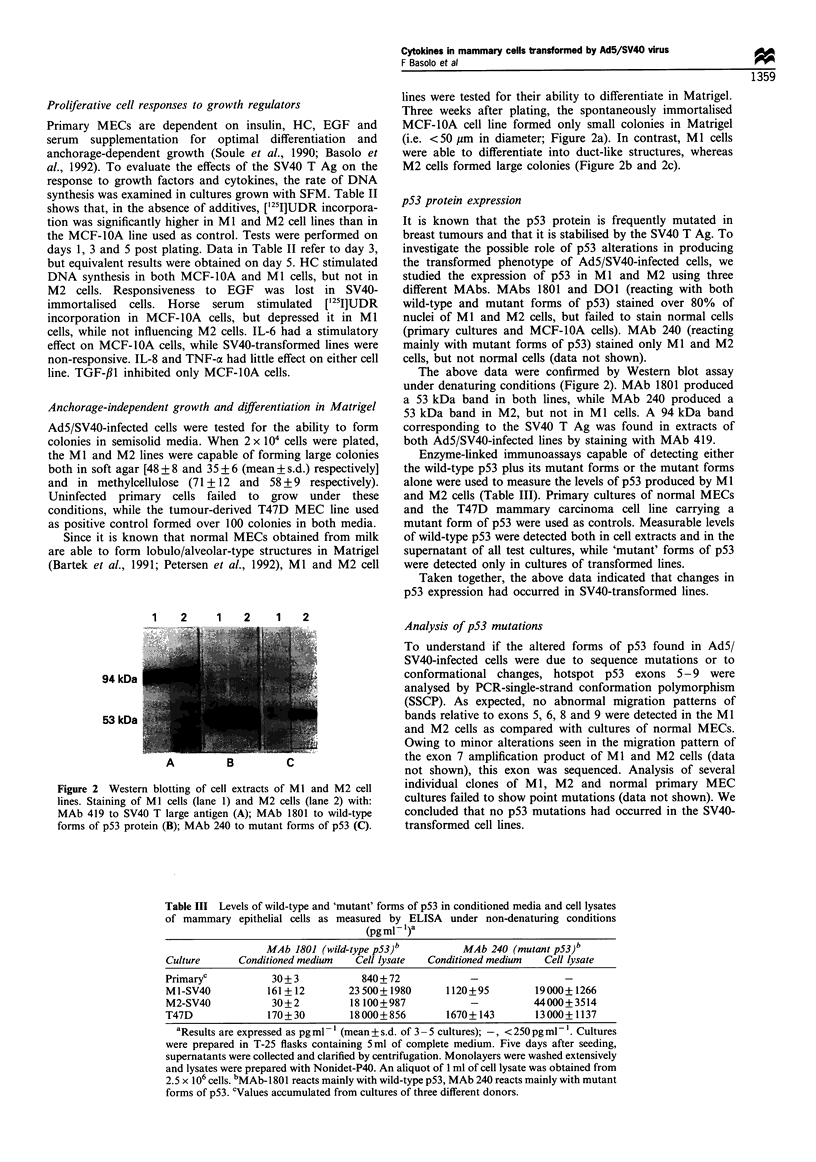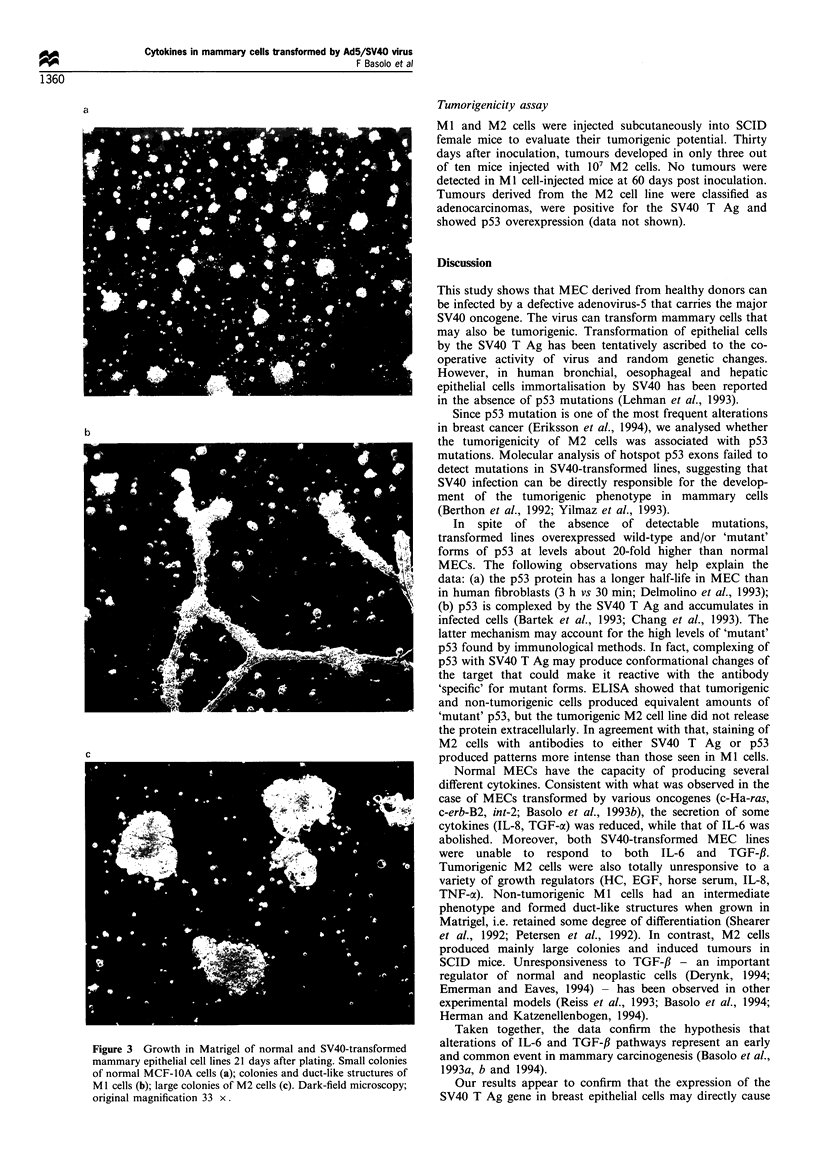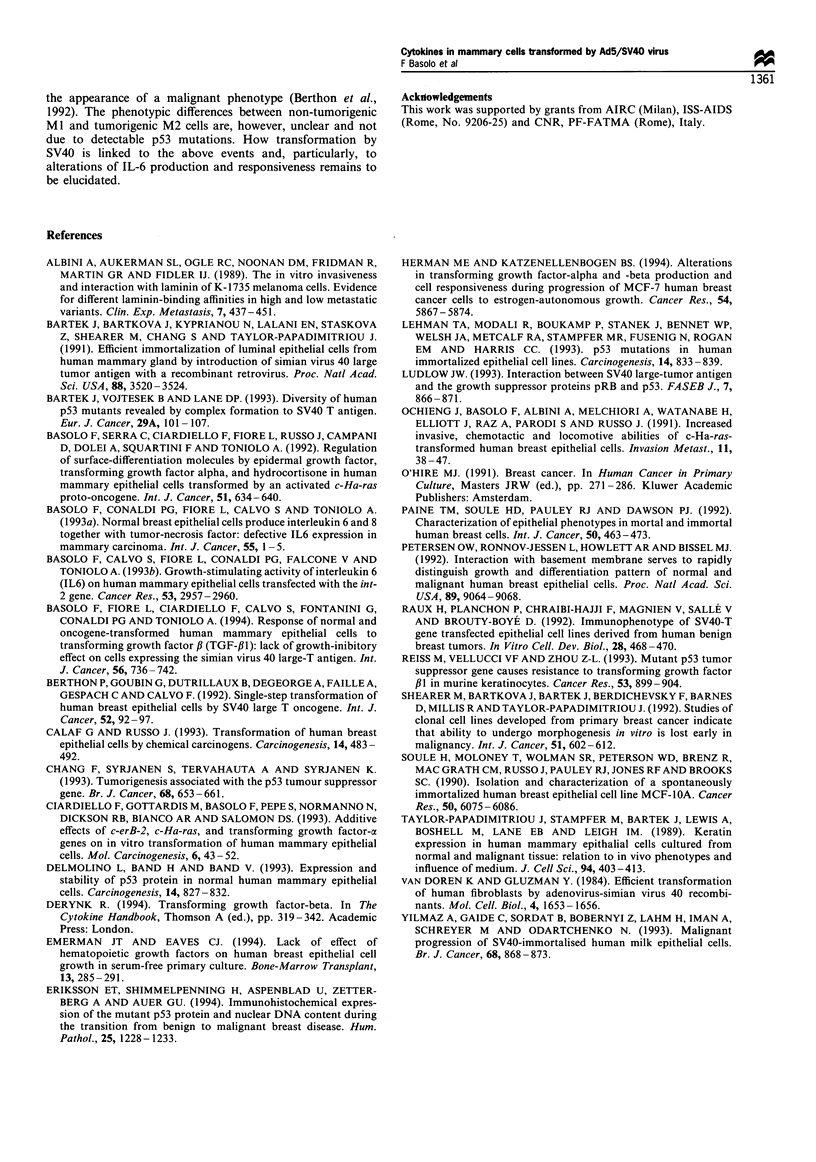# Defective interleukin six expression and responsiveness in human mammary cells transformed by an adeno 5/SV40 hybrid virus.

**DOI:** 10.1038/bjc.1996.258

**Published:** 1996-06

**Authors:** F. Basolo, L. Fiore, S. Calvo, V. Falcone, P. G. Conaldi, G. Fontanini, A. M. Caligo, G. Merlo, Y. Gluzman, A. Toniolo

**Affiliations:** Institute of Pathological Anatomy, University of Pisa, Italy.

## Abstract

**Images:**


British Journal of Cancer (1996) 73, 1356-1361
© 1996 Stockton Press All rights reserved 0007-0920/96 $12.00

### Defective interleukin six expression and responsiveness in human mammary cells transformed by an adeno 5/SV40 hybrid virus

F Basolo[1], L Fiore[1], S Calvo[1], V Falcone[2], PG Conaldi[2], G Fontanini[1], AM Caligo[1], G Merlo[3], Y Gluzman[4] and A Toniolo[2]

[1]*Institute of Pathological Anatomy, University of Pisa, Italy;* [2]*Institute of Medicine and Public Health, University of Pavia, Varese, Italy;* [3]*Friedrich Miescher-Institut, Basle, Switzerland;* [4]*Molecular Biology Section, Lederle Laboratories, Pearl River, NY, USA.*

**Summary** Mammary epithelial cells (MECs) were isolated and cultured from mammary glands of healthy women undergoing reduction mammoplasty. Normal MECs were infected with the transforming hybrid virus adeno-5/SV40. Two transformed epithelial cell lines, M1 and M2, were obtained, characterised phenotypically and studied for the production of and the response to cytokines and growth regulators. In both cell lines, expression of the SV40 large T antigen was associated with loss of interleukin 6 (IL-6) production and responsiveness as well as with down-regulation of IL-8 and transforming growth factor (TGF)-α production. Both M1 and M2 cell lines were capable of forming colonies in semisolid media, but upon injection into severe combined immunodeficient (SCID) mice only M2 cells were tumorigenic. DNA synthesis in M1 cells was partially inhibited by serum or TNF-α and weakly stimulated by hydrocortisone (HC) and IL-8. In contrast, M2 cells were totally unresponsive to a variety of growth regulators. Both lines overexpressed the p53 protein at levels about 20-fold higher than those observed in primary MEC cultures, but no mutations of the p53 gene could be detected. The data confirm the view that the expression in human mammary cells of different oncogenes – including the SV40 T antigen – is frequently associated with alterations of cytokine production and responsiveness.

**Keywords:** mammary cells; SV40; interleukin 6; cytokines

This study was initiated to investigate *in vitro* the early phenotypic alterations associated with the transforming activity of simian virus 40 (SV40) in human mammary epithelial cells (MECs) obtained from healthy donors. Several human breast cancer cell lines have already been obtained in different laboratories (O'Hire, 1991), but only a few human MEC lines have been derived from normal breast tissue (Soule *et al.*, 1990; Paine *et al.*, 1992). Owing to the extremely low frequency of spontaneous immortalisation, MEC lines have been established either by chemical treatment, by transfection with different oncogenes or by infection with oncogenic viruses (Calaf and Russo, 1993; Ciardiello *et al.*, 1993; Ochieng *et al.*, 1991; Berthon *et al.*, 1992). We used a replication-defective recombinant adeno-5/SV40 virus (Ad5/SV40) that carries the adenovirus type 5 capsid, the early genomic adenovirus region lacking the origin of replication, and the SV40 large T antigen (SV40 T Ag; Van Doren and Gluzman, 1984). Primary MEC cultures obtained from healthy donors undergoing reduction mammoplasty were infected with this transforming virus. The SV40 T Ag, which forms stable complexes with tumour-suppressor gene products p53 and Rb (Ludlow, 1993), appears to produce alterations of cytokine pathways similar to those observed in the neoplastic transformation of MECs by other oncogenes (Basolo *et al.*, 1993*a*,*b*). Of the two Ad5/SV40-immortalised MEC lines that were characterized, only one was tumorigenic when injected into immunodeficient mice. However, transformation of MECs by SV40 T Ag was consistently associated with changes in interleukin 6 (IL-6) expression and responsiveness.

### Materials and methods

### *Primary cultures of mammary epithelial cells and SV40 infection*

Normal breast tissue was obtained from healthy women undergoing reductive mammoplasty. Primary MECs were prepared by mechanic and collagenase dissociation and cultured in Dulbecco's modified Eagle medium (DMEM)/F12 low-calcium medium (0.04 mM $Ca^{2+}$) supplemented with insulin (5 μg $ml^{-1}$), hydrocortisone (HC; 500 ng $ml^{-1}$), human recombinant epidermal growth factor (EGF; 10 ng $ml^{-1}$), cholera toxin (100 ng $ml^{-1}$) and 5% horse serum, according to Soule's method (Soule *et al.*, 1990). Tissue culture reagents were from Sigma, (St. Louis, MO, USA). Pure MEC cultures were infected between 40 and 55 days post plating with a replication-incompetent $ori^-$ hybrid Ad5/SV40 virus (R4 strain; Van Doren and Gluzman, 1984) and grown in complete medium with standard calcium concentration (1.05 mM $Ca^{2+}$; high-calcium medium, HCM). After 20–25 days, colonies of transformed cells were picked up with the help of glass cylinders and expanded. MECs that were capable of growing over at least 15 passages and that were capable of forming colonies in semisolid media were considered transformed and further characterised. Two MEC lines originated from different donors (designated M1 and M2) were analysed in detail. Serum-free medium (SFM) was used to evaluate the response of MECs to growth regulators. SFM consisted of DMEM/F12 with 1.05 mM $Ca^{2+}$, 0.3% dialysed fetal bovine serum (FBS), 0.2% bovine serum albumin (BSA), human-transferrin (5 μg $ml^{-1}$), sodium-selenite (5 ng $ml^{-1}$) and insulin (10 μg $ml^{-1}$). Primary MEC cultures, the spontaneously immortalised MCF-10A normal breast cell line (Soule *et al.*, 1990) and the breast carcinoma-derived T47D cell line were used as controls.

### *Immunocytochemistry and immunoradiometric assay*

For immunocytochemical staining, cells were grown in chamber slides (Nunc, Naperville, IL, USA) and fixed in 1:1 (v/v) acetone–methanol at −20°C. The following antibodies were used: cytokeratin (K8, K14, K18 and K19; Unipath, Basingstoke, UK), p53 protein (MAbs 1801, 240, DO1, Oncogene Science, Manhasset, NY, USA) and SV40 T Ag (MAb 419, Oncogene Science). Cultures were stained with 0.1–1 μg of antibody in 100 μl of phosphate-buffered saline (PBS)–BSA. Reactivity was revealed by immunoperoxidase staining with an avidin–biotin complex kit (ABC kit; Vector, Burlinghame, CA, USA).

Correspondence: A Toniolo, University of Pavia, Institute of Medicine and Public Health, Viale Borri, 57–21100 Varese, Italy
Received 26 July 1995; revised 11 December 1995; accepted 20 December 1995

The expression of membrane antigens was measured with an immunoradiometric assay in live cells using MAbs to surface antigens as reported previously (Basolo *et al.*, 1992). The following MAbs were used: non-polymorphic HLA-A,B,C and HLA-DR (Technogenetics, Trezzano S/N, Italy), human milk fat globule Ag-1 and -2 (HMFG-1, -2; Oxoid Garbagnate Milanese, Italy); epithelial membrane antigen (EMA; BioGenex, San Ramon, CA, USA); transferrin receptor (CD71, Becton Dickinson, Milan, Italy); EGF receptor (EGF-R, extracellular domain; Oncogene Science).

*Cell response to growth regulators*

DNA synthesis was measured as incorporation of 5-[$^{125}$1000]-2′-Deoxyuridine ([$^{125}$I]UDR Amersham, Milan, Italy) by cells grown in SFM containing insulin (10 $\mu$g ml$^{-1}$). Cultures in 24-well plates were pulsed for 3 h with [$^{125}$I]UDR (2.5 × 10$^5$ c.p.m.) 1, 3 and 5 days post plating and processed as reported previously (Basolo *et al.*, 1994). The results are expressed as net c.p.m. per 50 000 cells. The influence of different growth regulators on DNA synthesis was studied: rh-IL-6 and rh-IL-8 (UBI, Lake Placid, NY, USA), rh-TNF-$\alpha$ and rh-EGF (Sigma), rh-TGF-$\beta$1 (R&D Systems, Minneapolis, MN, USA), HC (Sigma), horse serum (Gibco, Grand Island, NY, USA).

*Measurement of cytokine levels*

Conditioned media from both primary cultures and Ad5/SV40-infected cell lines were used to measure the release of IL-1-$\alpha$ and -$\beta$, IL-6, IL-8 and TGF-$\alpha$ with immunoenzyme assays [dosage kits were from: Genzyme, Boston, MA, USA (IL-1-$\alpha$; sensitivity 5 pg ml$^{-1}$; IL-1-$\beta$, sensitivity 1 pg ml$^{-1}$), R&D Systems, Minneapolis, MN, USA (IL-6, sensitivity 5 pg ml$^{-1}$; IL-8, sensitivity 30 pg ml$^{-1}$), Oncogene Science (TGF-$\alpha$, sensitivity 25 pg ml$^{-1}$)]. Samples were taken 3, 6 and 9 days after plating (reported data refer to day 6).

*Analysis of cytokine transcripts*

Cytokine-specific mRNAs were detected in total RNA extracted from 10$^6$ cells by the guanidinium thiocyanate method. After treatment with RNAase-free DNAase for 1 h at 37°C, cDNA was obtained by using M-MLV reverse transcriptase in conjunction with random hexamer primers (Clontech, Palo Alto, CA, USA). cDNA was then amplified by the polymerase chain reaction (PCR) using *Taq* polymerase and cytokine-specific primer pairs (Cytokine MAPPing Amplimers, Clontech). Amplification was carried out for the following human transcripts: IL-6 (amplified products 628 bp), IL-8 (amplified product 289 bp), TGF-$\alpha$ (amplified product 297 bp) and $\beta$-actin (amplified product 1126 bp). $\beta$-Actin was used as a control for mRNA detectability. Thirty amplification cycles were performed in a Hybaid thermal reactor (Hybaid, Teddington, UK). Amplification products were separated by electrophoresis on 2.5% agarose gels and visualised under UV light by staining with 0.5 $\mu$g ml$^{-1}$ ethidium bromide. ΦX174/*Hae*III digest was used as size marker.

*Detection of p53 protein by Western blotting and immunoenzyme assay*

Cell lysates were prepared with the following buffer: 150 mM sodium chloride, 50 mM Tris (pH 8.0), 5 mM EDTA, 1% Nonidet P-40, 1 mM phenylmethylsulphonyl fluoride, 20 $\mu$g ml$^{-1}$ aprotinin, and 25 $\mu$g ml$^{-1}$ leupeptin (30 min at 4°C; chemicals from Sigma). Protein extracts were resolved by sodium dodecyl sulphate (SDS)-polyacrylamide gel electrophoresis (8% acrylamide, 0.5% bis-acrylamide) and transferred to nitrocellulose paper by electroblotting (Bio-Rad, Milan, Italy). Duplicate membranes were incubated for 1 h in a blotting buffer containing 5% Carnation dried milk, followed by 15 h at 4°C in Tris-borate buffer containing 1 $\mu$g ml$^{-1}$ of anti-p53 MAbs (MAbs 1801, DO1, and 240). MAbs 1801 and DO1 react preferentially with the C-terminal portion of the wild-type protein, but also with mutant forms, whereas MAb 240 recognises mainly the tertiary structure of several mutant forms of p53. The reaction was revealed by immunoperoxidase staining (Vector).

Levels of wild-type and mutant forms of the p53 protein were measured in culture supernatants and in cell extracts prepared as above. Two ELISAs (Oncogene Science) were used: pantropic p53 (MAb 1801; sensitivity 10 pg ml$^{-1}$) and mutant-selective p53 (MAb 240; sensitivity 250 pg ml$^{-1}$). A polyclonal rabbit antibody to p53 and peroxidase-conjugated goat anti-rabbit IgG allowed antigen determination.

*Analysis of p53 mutations*

A human p53 amplimer panel (Clontech) allowed the PCR amplification of exons 5, 6, 7, 8 and 9. Template DNA (100 ng) was subjected to 34 amplification cycles in 10 $\mu$l of reaction mixture containing: 1 × PCR buffer (50 mM potassium chloride, 10 mM Tris, 1.5 mM magnesium chloride), 200 $\mu$M each dNTPs, 3–10 pmol of each primer, 2.5 U of *Taq* polymerase and 0.5 $\mu$l of [$^{32}$P]dCTP (3000 Ci mmol$^{-1}$; Amersham, Milan, Italy). Aliquots of 1 $\mu$l of each PCR product were diluted with 4 $\mu$l of SSCP-loading buffer (95% formamide, 20 mM EDTA, 0.05% bromophenol blue, 0.05% xylene cyanole), denatured 5 min at 95°C, loaded onto 6% polyacrylamide–bis-acrylamide gel (49:1), 5% glycerol, 0.5 × TBE and run for 3 h at 15 w. Bands were detected by autoradiography on Kodak X-AR 5 film.

The entire exon 7 of the transformed M1 and M2 lines and of a normal primary MEC culture was amplified by PCR. Specific oligonucleotide primers were designed from the flanking intronic sequences: upstream (1775) 5′-CGCGCACTGGCCTCATCTT-3′; downstream (2253) 5′-TCAGCGGCAAGCAGAGGCTG-3′. The *Bam*HI and *Hin*dIII restriction sites were added to, respectively, the upstream and the downstream primer to facilitate subcloning. Thirty amplification cycles were performed and PCR products were ethanol precipitated, resuspended in 10 mM Tris pH 7.5, digested with *Bam*HI and *Hin*dIII enzymes, purified by 1.5% agarose electrophoresis, eluted and ligated into *Bam*HI/*Hin*dIII-digested pBluescript SKII (Stratagene, La Jolla, CA, USA). Recombinant clones were obtained and checked by restriction analysis. Sequencing was performed on 5 $\mu$g of purified plasmid DNA using the T3 primer (Boehringer, Mannheim, Germany) with the Sequenase 2.0 system (US Biochemicals, Cleveland, OH, USA).

*Anchorage-independent growth and Matrigel colonisation assay*

For colony-formation in soft agar and methylcellulose, 2 × 10$^4$ cells were suspended in 0.5 ml of HCM supplemented with either 0.3% Agar Noble (Difco, Detroit, MI, USA) or 0.8% carboxymethylcellulose (Sigma) and layered on a 0.8% Agar Noble base (0.25 ml in 2 cm$^2$ wells). Twenty-one days post plating, colonies ⩾60 $\mu$m in diameter were photographed and counted.

The Matrigel colonisation assay was performed as described previously (Albini, 1989). Matrigel (0.5 ml at 10 mg ml$^{-1}$; Collaborative Research, Bedford, MA, USA) was polymerised at 37°C for 1 h in wells of a 24-well plate. Cells suspended in HCM were seeded at 5 × 10$^4$ onto the top of the gel. After 1, 2 and 3 weeks incubation, cultures were observed for morphology and photographed.

*Tumorigenicity assay*

Cells cultured in HCM were detached by trypsinisation and washed twice in PBS–1% BSA. Approximately 1 million and 10 million cells suspended in 0.2 ml of Matrigel were injected subcutaneously into the left and right flanks of female severe combined immunodeficient (SCID) mice (IFFA-Credo, Milan, Italy). The animals were maintained in laminar flow cabinets and given sterile food and acidic water *ad libitum*. The sites of injection were inspected weekly.

### Results

*Phenotypic characterisation of Ad5/SV40-infected breast cell lines*

After Ad5/SV40 infection, MECs were maintained in HCM. Uninfected cultures began to senesce after 2–4 passages. In contrast, 20–30 days after infection, colonies appeared in cultures exposed to Ad5/SV40. Colonies were expanded and passaged serially. At the present time, both lines have been in culture for over 2 years (i.e. over 300 population doublings). On passages 10–15 the cells appeared to stop growing, but 3–5 weeks later focal growth resumed. Cells of the M1 and M2 lines were larger than uninfected cells and tended to grow to higher densities. Transformed lines formed domes similar to those produced by the spontaneously immortalised MCF-10A MEC line (Soule *et al.*, 1990). To see whether SV40 immortalisation could influence the expression of MEC antigens, cells were reacted with selected MAbs to both surface (immunoradiometric assay) and intracellular antigens (immunostaining; data not shown). Transformed cells were essentially indistinguishable from normal MECs (Basolo *et al.*, 1992). They expressed HLA class I, but not class II, molecules, were positive for epithelial markers such as EMA and HMFG-1/-2 as well as for EGF and transferrin receptors. Both lines expressed cytokeratins 8, 14 and 18 in the absence of cytokeratin 19, a finding in agreement with previous results obtained in cultured MECs (Taylor-Papadimitriou *et al.*, 1989; Paine *et al.*, 1992; Raux *et al.*, 1992). M1 and M2 lines showed strong nuclear reactivity for the SV40 T Ag (over 95% positive cells).

*Production of cytokines*

Conditioned media from primary MEC cultures and from transformed lines (M1, M2, and T47D) were tested for the presence of IL-1-α/β, IL-6, IL-8 and TGF-α (Table I). As previously reported (Basolo *et al.*, 1993*a*), no culture released IL-1-α or -β, whereas primary MEC cultures produced high levels of IL-6, IL-8, and TGF-α. The M1, M2 and T47D cell lines were unable to release IL-6 and secreted reduced levels of both IL-8 and TGF-α. Analysis of mRNA transcripts confirmed that IL-6 expression was abolished in transformed cells (Figure 1).

**Figure 1** PCR amplification of total cellular RNA obtained from the SV40-transformed M1 and M2 cell lines and from a control culture of normal mammary epithelial cells. Primers specific for TGF-α, IL-6 and IL-8 were used. Ethidium bromide-stained agarose gel. Lane 1, M1 cells; lane 2, M2 cells; lane 3, primary culture no. 25; lane 4, positive control. Molecular size markers are shown on the left.

**Table I** Levels of cytokines released in the medium by normal mammary epithelial cells, SV40-transformed M1 and M2 cell lines and tumour-derived T47D cell line ($pg\,ml^{-1}$)[a]

| *Cytokine* | *Primary cultures*[b] | *M1* | *M2* | *T47D* |
|---|---|---|---|---|
| IL-1-α | – | – | – | – |
| IL-1β | – | – | – | – |
| IL-6 | 2280±320 | – | – | – |
| IL-8 | 2150±240 | 45±28 | 50±42 | 64±40 |
| TGF-α | 158±35 | 56±18 | 68±32 | 47±28 |

[a]Cytokine concentrations were measured by enzyme-linked immunoassay (mean±s.d. of 3–5 cultures). Conditioned media were collected 6 days after plating. –, below the detection limit. [b] Values accumulated from cultures of three different donors.

**Table II** Incorporation of [$^{125}$I]UDR in the MCF-10A control cell line in SV40-transformed M1 and M2 cell lines stimulated with different growth regulators[a]

| *Additives* | *MCF-10A* | *M1* | *M2* |
|---|---|---|---|
| None | 991±82 | 2937±31 | 8459±743 |
| HC (100 $ng\,ml^{-1}$) | 2699±134* | 4892±415* | 8909±1141 |
| EGF (10 $ng\,ml^{-1}$) | 5946±187* | 3270±245 | 9494±1512 |
| Horse serum (5%) | 4668±415* | 786±36* | 9957±2333 |
| IL-6 (10 $ng\,ml^{-1}$) | 1825±124* | 3160±96 | 8972±648 |
| IL-8 (10 $ng\,ml^{-1}$) | 1110±56 | 4415±50* | 9637±937 |
| TNF-α (10 $ng\,ml^{-1}$) | 571±52* | 2060±63* | 8661±863 |
| TGF-β (5 $ng\,ml^{-1}$) | 442±38* | 2909±223 | 10 512±1304 |

[a]Cultures in serum-free medium containing insulin (10 $\mu g\,ml^{-1}$) were stimulated with additives for 3 days and pulsed with [$^{125}$I]UDR for 3 h. Values are reported as c.p.m./50 000 cells; mean±s.d. of 3–5 cultures. Asterisks refer to values that are significantly different from those obtained in control cultures given no additives ($P<0.001$).

*Proliferative cell responses to growth regulators*

Primary MECs are dependent on insulin, HC, EGF and serum supplementation for optimal differentiation and anchorage-dependent growth (Soule *et al.*, 1990; Basolo *et al.*, 1992). To evaluate the effects of the SV40 T Ag on the response to growth factors and cytokines, the rate of DNA synthesis was examined in cultures grown with SFM. Table II shows that, in the absence of additives, [$^{125}$I]UDR incorporation was significantly higher in M1 and M2 cell lines than in the MCF-10A line used as control. Tests were performed on days 1, 3 and 5 post plating. Data in Table II refer to day 3, but equivalent results were obtained on day 5. HC stimulated DNA synthesis in both MCF-10A and M1 cells, but not in M2 cells. Responsiveness to EGF was lost in SV40-immortalised cells. Horse serum stimulated [$^{125}$I]UDR incorporation in MCF-10A cells, but depressed it in M1 cells, while not influencing M2 cells. IL-6 had a stimulatory effect on MCF-10A cells, while SV40-transformed lines were non-responsive. IL-8 and TNF-$\alpha$ had little effect on either cell line. TGF-$\beta$1 inhibited only MCF-10A cells.

*Anchorage-independent growth and differentiation in Matrigel*

Ad5/SV40-infected cells were tested for the ability to form colonies in semisolid media. When $2\times10^4$ cells were plated, the M1 and M2 lines were capable of forming large colonies both in soft agar [48 ± 8 and 35 ± 6 (mean ± s.d.) respectively] and in methylcellulose (71 ± 12 and 58 ± 9 respectively). Uninfected primary cells failed to grow under these conditions, while the tumour-derived T47D MEC line used as positive control formed over 100 colonies in both media.

Since it is known that normal MECs obtained from milk are able to form lobulo/alveolar-type structures in Matrigel (Bartek *et al.*, 1991; Petersen *et al.*, 1992), M1 and M2 cell lines were tested for their ability to differentiate in Matrigel. Three weeks after plating, the spontaneously immortalised MCF-10A cell line formed only small colonies in Matrigel (i.e. < 50 μm in diameter; Figure 2a). In contrast, M1 cells were able to differentiate into duct-like structures, whereas M2 cells formed large colonies (Figure 2b and 2c).

**Figure 2** Western blotting of cell extracts of M1 and M2 cell lines. Staining of M1 cells (lane 1) and M2 cells (lane 2) with: MAb 419 to SV40 T large antigen (**A**); MAb 1801 to wild-type forms of p53 protein (**B**); MAb 240 to mutant forms of p53 (**C**).

*p53 protein expression*

It is known that the p53 protein is frequently mutated in breast tumours and that it is stabilised by the SV40 T Ag. To investigate the possible role of p53 alterations in producing the transformed phenotype of Ad5/SV40-infected cells, we studied the expression of p53 in M1 and M2 using three different MAbs. MAbs 1801 and DO1 (reacting with both wild-type and mutant forms of p53) stained over 80% of nuclei of M1 and M2 cells, but failed to stain normal cells (primary cultures and MCF-10A cells). MAb 240 (reacting mainly with mutant forms of p53) stained only M1 and M2 cells, but not normal cells (data not shown).

The above data were confirmed by Western blot assay under denaturing conditions (Figure 2). MAb 1801 produced a 53 kDa band in both lines, while MAb 240 produced a 53 kDa band in M2, but not in M1 cells. A 94 kDa band corresponding to the SV40 T Ag was found in extracts of both Ad5/SV40-infected lines by staining with MAb 419.

Enzyme-linked immunoassays capable of detecting either the wild-type p53 plus its mutant forms or the mutant forms alone were used to measure the levels of p53 produced by M1 and M2 cells (Table III). Primary cultures of normal MECs and the T47D mammary carcinoma cell line carrying a mutant form of p53 were used as controls. Measurable levels of wild-type p53 were detected both in cell extracts and in the supernatant of all test cultures, while 'mutant' forms of p53 were detected only in cultures of transformed lines.

Taken together, the above data indicated that changes in p53 expression had occurred in SV40-transformed lines.

*Analysis of p53 mutations*

To understand if the altered forms of p53 found in Ad5/SV40-infected cells were due to sequence mutations or to conformational changes, hotspot p53 exons 5–9 were analysed by PCR-single-strand conformation polymorphism (SSCP). As expected, no abnormal migration patterns of bands relative to exons 5, 6, 8 and 9 were detected in the M1 and M2 cells as compared with cultures of normal MECs. Owing to minor alterations seen in the migration pattern of the exon 7 amplification product of M1 and M2 cells (data not shown), this exon was sequenced. Analysis of several individual clones of M1, M2 and normal primary MEC cultures failed to show point mutations (data not shown). We concluded that no p53 mutations had occurred in the SV40-transformed cell lines.

**Table III** Levels of wild-type and 'mutant' forms of p53 in conditioned media and cell lysates of mammary epithelial cells as measured by ELISA under non-denaturing conditions (pg ml$^{-1}$)[a]

| | *MAb 1801 (wild-type p53)*[b] | | *MAb 240 (mutant p53)*[b] | |
|---|---|---|---|---|
| *Culture* | *Conditioned medium* | *Cell lysate* | *Conditioned medium* | *Cell lysate* |
| Primary[c] | 30 ± 3 | 840 ± 72 | – | – |
| M1-SV40 | 161 ± 12 | 23 500 ± 1980 | 1120 ± 95 | 19 000 ± 1266 |
| M2-SV40 | 30 ± 2 | 18 100 ± 987 | – | 44 000 ± 3514 |
| T47D | 170 ± 30 | 18 000 ± 856 | 1670 ± 143 | 13 000 ± 1137 |

[a]Results are expressed as pg ml$^{-1}$ (mean ± s.d. of 3–5 cultures); –, < 250 pg ml$^{-1}$. Cultures were prepared in T-25 flasks containing 5 ml of complete medium. Five days after seeding, supernatants were collected and clarified by centrifugation. Monolayers were washed extensively and lysates were prepared with Nonidet-P40. An aliquot of 1 ml of cell lysate was obtained from $2.5\times10^6$ cells. [b]MAb-1801 reacts mainly with wild-type p53, MAb 240 reacts mainly with mutant forms of p53. [c]Values accumulated from cultures of three different donors.

Figure 3 Growth in Matrigel of normal and SV40-transformed mammary epithelial cell lines 21 days after plating. Small colonies of normal MCF-10A cells (a); colonies and duct-like structures of M1 cells (b); large colonies of M2 cells (c). Dark-field microscopy; original magnification 33 ×.

*Tumorigenicity assay*

M1 and M2 cells were injected subcutaneously into SCID female mice to evaluate their tumorigenic potential. Thirty days after inoculation, tumours developed in only three out of ten mice injected with $10^7$ M2 cells. No tumours were detected in M1 cell-injected mice at 60 days post inoculation. Tumours derived from the M2 cell line were classified as adenocarcinomas, were positive for the SV40 T Ag and showed p53 overexpression (data not shown).

### Discussion

This study shows that MEC derived from healthy donors can be infected by a defective adenovirus-5 that carries the major SV40 oncogene. The virus can transform mammary cells that may also be tumorigenic. Transformation of epithelial cells by the SV40 T Ag has been tentatively ascribed to the co-operative activity of virus and random genetic changes. However, in human bronchial, oesophageal and hepatic epithelial cells immortalisation by SV40 has been reported in the absence of p53 mutations (Lehman *et al.*, 1993).

Since p53 mutation is one of the most frequent alterations in breast cancer (Eriksson *et al.*, 1994), we analysed whether the tumorigenicity of M2 cells was associated with p53 mutations. Molecular analysis of hotspot p53 exons failed to detect mutations in SV40-transformed lines, suggesting that SV40 infection can be directly responsible for the development of the tumorigenic phenotype in mammary cells (Berthon *et al.*, 1992; Yilmaz *et al.*, 1993).

In spite of the absence of detectable mutations, transformed lines overexpressed wild-type and/or 'mutant' forms of p53 at levels about 20-fold higher than normal MECs. The following observations may help explain the data: (a) the p53 protein has a longer half-life in MEC than in human fibroblasts (3 h *vs* 30 min; Delmolino *et al.*, 1993); (b) p53 is complexed by the SV40 T Ag and accumulates in infected cells (Bartek *et al.*, 1993; Chang *et al.*, 1993). The latter mechanism may account for the high levels of 'mutant' p53 found by immunological methods. In fact, complexing of p53 with SV40 T Ag may produce conformational changes of the target that could make it reactive with the antibody 'specific' for mutant forms. ELISA showed that tumorigenic and non-tumorigenic cells produced equivalent amounts of 'mutant' p53, but the tumorigenic M2 cell line did not release the protein extracellularly. In agreement with that, staining of M2 cells with antibodies to either SV40 T Ag or p53 produced patterns more intense than those seen in M1 cells.

Normal MECs have the capacity of producing several different cytokines. Consistent with what was observed in the case of MECs transformed by various oncogenes (c-Ha-*ras*, c-*erb*-B2, *int*-2; Basolo *et al.*, 1993*b*), the secretion of some cytokines (IL-8, TGF-$\alpha$) was reduced, while that of IL-6 was abolished. Moreover, both SV40-transformed MEC lines were unable to respond to both IL-6 and TGF-$\beta$. Tumorigenic M2 cells were also totally unresponsive to a variety of growth regulators (HC, EGF, horse serum, IL-8, TNF-$\alpha$). Non-tumorigenic M1 cells had an intermediate phenotype and formed duct-like structures when grown in Matrigel, i.e. retained some degree of differentiation (Shearer *et al.*, 1992; Petersen *et al.*, 1992). In contrast, M2 cells produced mainly large colonies and induced tumours in SCID mice. Unresponsiveness to TGF-$\beta$ – an important regulator of normal and neoplastic cells (Derynk, 1994; Emerman and Eaves, 1994) – has been observed in other experimental models (Reiss *et al.*, 1993; Basolo *et al.*, 1994; Herman and Katzenellenbogen, 1994).

Taken together, the data confirm the hypothesis that alterations of IL-6 and TGF-$\beta$ pathways represent an early and common event in mammary carcinogenesis (Basolo *et al.*, 1993*a*, *b* and 1994).

Our results appear to confirm that the expression of the SV40 T Ag gene in breast epithelial cells may directly cause the appearance of a malignant phenotype (Berthon *et al.*, 1992). The phenotypic differences between non-tumorigenic M1 and tumorigenic M2 cells are, however, unclear and not due to detectable p53 mutations. How transformation by SV40 is linked to the above events and, particularly, to alterations of IL-6 production and responsiveness remains to be elucidated.

**Acknowledgements**
This work was supported by grants from AIRC (Milan), ISS-AIDS (Rome, No. 9206-25) and CNR, PF-FATMA (Rome), Italy.

### References

ALBINI A, AUKERMAN SL, OGLE RC, NOONAN DM, FRIDMAN R, MARTIN GR AND FIDLER IJ. (1989). The in vitro invasiveness and interaction with laminin of K-1735 melanoma cells. Evidence for different laminin-binding affinities in high and low metastatic variants. *Clin. Exp. Metastasis*, **7**, 437–451.

BARTEK J, BARTKOVA J, KYPRIANOU N, LALANI EN, STASKOVA Z, SHEARER M, CHANG S AND TAYLOR-PAPADIMITRIOU J. (1991). Efficient immortalization of luminal epithelial cells from human mammary gland by introduction of simian virus 40 large tumor antigen with a recombinant retrovirus. *Proc. Natl Acad. Sci. USA*, **88**, 3520–3524.

BARTEK J, VOJTESEK B AND LANE DP. (1993). Diversity of human p53 mutants revealed by complex formation to SV40 T antigen. *Eur. J. Cancer*, **29A**, 101–107.

BASOLO F, SERRA C, CIARDIELLO F, FIORE L, RUSSO J, CAMPANI D, DOLEI A, SQUARTINI F AND TONIOLO A. (1992). Regulation of surface-differentiation molecules by epidermal growth factor, transforming growth factor alpha, and hydrocortisone in human mammary epithelial cells transformed by an activated *c-Ha-ras* proto-oncogene. *Int. J. Cancer*, **51**, 634–640.

BASOLO F, CONALDI PG, FIORE L, CALVO S AND TONIOLO A. (1993*a*). Normal breast epithelial cells produce interleukin 6 and 8 together with tumor-necrosis factor: defective IL6 expression in mammary carcinoma. *Int. J. Cancer*, **55**, 1–5.

BASOLO F, CALVO S, FIORE L, CONALDI PG, FALCONE V AND TONIOLO A. (1993*b*). Growth-stimulating activity of interleukin 6 (IL6) on human mammary epithelial cells transfected with the *int*-2 gene. *Cancer Res.*, **53**, 2957–2960.

BASOLO F, FIORE L, CIARDIELLO F, CALVO S, FONTANINI G, CONALDI PG AND TONIOLO A. (1994). Response of normal and oncogene-transformed human mammary epithelial cells to transforming growth factor $\beta$ (TGF-$\beta$1): lack of growth-inibitory effect on cells expressing the simian virus 40 large-T antigen. *Int. J. Cancer*, **56**, 736–742.

BERTHON P, GOUBIN G, DUTRILLAUX B, DEGEORGE A, FAILLE A, GESPACH C AND CALVO F. (1992). Single-step transformation of human breast epithelial cells by SV40 large T oncogene. *Int. J. Cancer*, **52**, 92–97.

CALAF G AND RUSSO J. (1993). Transformation of human breast epithelial cells by chemical carcinogens. *Carcinogenesis*, **14**, 483–492.

CHANG F, SYRJANEN S, TERVAHAUTA A AND SYRJANEN K. (1993). Tumorigenesis associated with the p53 tumour suppressor gene. *Br. J. Cancer*, **68**, 653–661.

CIARDIELLO F, GOTTARDIS M, BASOLO F, PEPE S, NORMANNO N, DICKSON RB, BIANCO AR AND SALOMON DS. (1993). Additive effects of *c-erB-2*, *c-Ha-ras*, and transforming growth factor-$\alpha$ genes on in vitro transformation of human mammary epithelial cells. *Mol. Carcinogenesis*, **6**, 43–52.

DELMOLINO L, BAND H AND BAND V. (1993). Expression and stability of p53 protein in normal human mammary epithelial cells. *Carcinogenesis*, **14**, 827–832.

DERYNK R. (1994). Transforming growth factor-beta. In *The Cytokine Handbook*, Thomson A (ed.), pp. 319–342. Academic Press: London.

EMERMAN JT AND EAVES CJ. (1994). Lack of effect of hematopoietic growth factors on human breast epithelial cell growth in serum-free primary culture. *Bone-Marrow Transplant*, **13**, 285–291.

ERIKSSON ET, SHIMMELPENNING H, ASPENBLAD U, ZETTERBERG A AND AUER GU. (1994). Immunohistochemical expression of the mutant p53 protein and nuclear DNA content during the transition from benign to malignant breast disease. *Hum. Pathol.*, **25**, 1228–1233.

HERMAN ME AND KATZENELLENBOGEN BS. (1994). Alterations in transforming growth factor-alpha and -beta production and cell responsiveness during progression of MCF-7 human breast cancer cells to estrogen-autonomous growth. *Cancer Res.*, **54**, 5867–5874.

LEHMAN TA, MODALI R, BOUKAMP P, STANEK J, BENNET WP, WELSH JA, METCALF RA, STAMPFER MR, FUSENIG N, ROGAN EM AND HARRIS CC. (1993). p53 mutations in human immortalized epithelial cell lines. *Carcinogenesis*, **14**, 833–839.

LUDLOW JW. (1993). Interaction between SV40 large-tumor antigen and the growth suppressor proteins pRB and p53. *FASEB J.*, **7**, 866–871.

OCHIENG J, BASOLO F, ALBINI A, MELCHIORI A, WATANABE H, ELLIOTT J, RAZ A, PARODI S AND RUSSO J. (1991). Increased invasive, chemotactic and locomotive abilities of c-Ha-*ras*-transformed human breast epithelial cells. *Invasion Metast.*, **11**, 38–47.

O'HIRE MJ. (1991). Breast cancer. In *Human Cancer in Primary Culture*, Masters JRW (ed.), pp. 271–286. Kluwer Academic Publishers: Amsterdam.

PAINE TM, SOULE HD, PAULEY RJ AND DAWSON PJ. (1992). Characterization of epithelial phenotypes in mortal and immortal human breast cells. *Int. J. Cancer*, **50**, 463–473.

PETERSEN OW, RONNOV-JESSEN L, HOWLETT AR AND BISSEL MJ. (1992). Interaction with basement membrane serves to rapidly distinguish growth and differentiation pattern of normal and malignant human breast epithelial cells. *Proc. Natl Acad. Sci. USA*, **89**, 9064–9068.

RAUX H, PLANCHON P, CHRAIBI-HAJJI F, MAGNIEN V, SALLÉ V AND BROUTY-BOYÉ D. (1992). Immunophenotype of SV40-T gene transfected epithelial cell lines derived from human benign breast tumors. *In Vitro Cell. Dev. Biol.*, **28**, 468–470.

REISS M, VELLUCCI VF AND ZHOU Z-L. (1993). Mutant p53 tumor suppressor gene causes resistance to transforming growth factor $\beta$1 in murine keratinocytes. *Cancer Res.*, **53**, 899–904.

SHEARER M, BARTKOVA J, BARTEK J, BERDICHEVSKY F, BARNES D, MILLIS R AND TAYLOR-PAPADIMITRIOU J. (1992). Studies of clonal cell lines developed from primary breast cancer indicate that ability to undergo morphogenesis *in vitro* is lost early in malignancy. *Int. J. Cancer*, **51**, 602–612.

SOULE H, MOLONEY T, WOLMAN SR, PETERSON WD, BRENZ R, MAC GRATH CM, RUSSO J, PAULEY RJ, JONES RF AND BROOKS SC. (1990). Isolation and characterization of a spontaneously immortalized human breast epithelial cell line MCF-10A. *Cancer Res.*, **50**, 6075–6086.

TAYLOR-PAPADIMITRIOU J, STAMPFER M, BARTEK J, LEWIS A, BOSHELL M, LANE EB AND LEIGH IM. (1989). Keratin expression in human mammary epithalial cells cultured from normal and malignant tissue: relation to in vivo phenotypes and influence of medium. *J. Cell Sci.*, **94**, 403–413.

VAN DOREN K AND GLUZMAN Y. (1984). Efficient transformation of human fibroblasts by adenovirus-simian virus 40 recombinants. *Mol. Cell. Biol.*, **4**, 1653–1656.

YILMAZ A, GAIDE C, SORDAT B, BOBERNYI Z, LAHM H, IMAN A, SCHREYER M AND ODARTCHENKO N. (1993). Malignant progression of SV40-immortalised human milk epithelial cells. *Br. J. Cancer*, **68**, 868–873.